# Self-Reported Gastrointestinal Symptoms Associated with NSAIDs and Caffeine Consumption in a Jordanian Subpopulation

**DOI:** 10.3390/medicina60091519

**Published:** 2024-09-18

**Authors:** Sofian Al Shboul, Omar Maloul, Hamza Al-Trad, Yazan Maloul, Wa’ed AlHarahsheh, Doa’a Mosallam, Sondos Al-Sarayreh, Rania AlRashaydah, Aya AlSarayreh, Ashraf I. Khasawneh, Tareq Saleh

**Affiliations:** 1 Department of Pharmacology and Public Health, Faculty of Medicine, The Hashemite University, Zarqa 13133, Jordan; 1837097@std.hu.edu.jo (O.M.); 1834015@std.hu.edu.jo (H.A.-T.); 1939207@std.hu.edu.jo (Y.M.); 1833923@std.hu.edu.jo (W.A.); 1834414@std.hu.edu.jo (D.M.); 1834582@std.hu.edu.jo (S.A.-S.); 1833872@std.hu.edu.jo (R.A.); 1831533@std.hu.edu.jo (A.A.); 2 Department of Microbiology, Pathology, and Forensic Medicine, Faculty of Medicine, The Hashemite University, Zarqa 13133, Jordan; ashraf.khasawneh@nih.gov; 3 Department of Laboratory Medicine, National Institutes of Health Clinical Center, Bethesda, MD 20892, USA

**Keywords:** peptic ulcer disease, Jordan, gastrointestinal, NSAIDs, caffeine consumption

## Abstract

*Background:* Nonsteroidal anti-inflammatory drugs (NSAIDs) and caffeine-containing beverages are widely consumed but their impact on gastrointestinal (GI) health requires further investigation. This cross-sectional study investigated the relationship between NSAIDs use, caffeinated drink consumption, and the prevalence of self-reported GI symptoms in a Jordanian subpopulation. *Methods:* An online survey was administered to 400 Jordanian individuals aged 18–65 years. Data on sociodemographics, NSAIDs use, caffeine consumption, peptic ulcer disease (PUD) history, and GI symptoms were collected. Contingency tables were used to calculate odds ratios (ORs) and 95% confidence intervals (CIs) for the associations between exposures and outcomes. *Results:* The prevalence of self-reported PUD-related GI symptoms was 6.0%. NSAID users had higher odds of PUD (OR = 2.431) and related GI symptoms, including abdominal pain (OR = 4.688, *p* < 0.001) and discomfort (OR = 8.068, *p* < 0.001). Caffeine consumption was associated with self-reported burning stomach pain (OR = 14.104, *p* < 0.001), fullness (OR = 8.304, *p* = 0.010), and bloating (OR = 8.304, *p* = 0.010). Coffee, tea, soft drinks, and energy drinks were associated with increased odds of various GI symptoms (ORs 2.018-12.715, *p* < 0.05). *Conclusions:* NSAIDs use and caffeine consumption were independently associated with the increased prevalence of self-reported PUD and related GI symptoms. Despite the lack of adjustment for necessary confounders, our findings highlight the importance of considering the potential GI effects of NSAIDs and caffeine. Public health strategies promoting their safe use may help reduce the burden of GI disorders.

## 1. Introduction

Peptic ulcers, characterized by open lesions on the inner lining of the stomach or upper small intestine, are a significant global health burden [1]. The prevalence of peptic ulcers varies worldwide, with estimates ranging from 5 to 10% in the general population [2]. Non-steroidal anti-inflammatory drugs (NSAIDs) and caffeine-containing drinks have been implicated in the development and exacerbation of peptic ulcers [3,4], but their precise roles remain complex.

NSAIDs, including ibuprofen and naproxen, are widely used analgesics, anti-inflammatories, and antipyretics [5]. These agents exert their therapeutic effects by inhibiting cyclooxygenase (COX) enzymes, namely, COX-1 and COX-2, which are essential for the conversion of arachidonic acid (AA) into various inflammatory mediators such as prostaglandins and prostacyclins [6,7,8]. COX-1 activity is critical for protecting the stomach and intestinal epithelium, while COX-2 is induced by inflammatory stimuli and contributes to tissue repair and wound healing [9,10]. NSAIDs are classified based on their selectivity for COX-1 and/or COX-2, with agents like aspirin, ibuprofen, and diclofenac being more selective for COX-1, providing cardioprotection but with serious GI side effects. In contrast, COX-2 selective inhibitors like celecoxib and etoricoxib have opposite effects on the cardiovascular and GI systems. Prolonged NSAIDs use is associated with various adverse effects, including GI complications, cardiovascular risks, renal dysfunction, and liver issues [5,11]. Unfortunately, most NSAIDs are available for consumption over the counter in Jordan, and previous reports have indicated a relatively higher use rate among Jordanians with poor knowledge of their potential adverse effects [1,2,3].

Caffeine and related xanthine derivatives, such as theobromine and theophylline, are naturally found in various food and drink sources, including coffee, tea, cocoa, and soft drinks [12]. These compounds have been reported to increase fatty acid secretion and stimulate gastric acid production, potentially contributing to the erosion of the gastric mucosa and predisposing individuals to ulceration [13,14,15]. Additionally, caffeine has been shown to enhance gastric motility, which may exacerbate existing ulcers or delay the healing process [16]. However, the impact of caffeine intake on ulcer development varies among individuals and can be influenced by factors such as dosage, frequency of consumption, and concomitant use of ulcerogenic agents like NSAIDs.

Despite the known risks associated with NSAIDs and caffeine-containing drinks, there is limited information on their combined effects on peptic ulcer disease (PUD) prevalence, particularly in specific populations where the risk of their concomitant use is relatively higher. This cross-sectional study aims to investigate the relationship between NSAIDs use, caffein drinks consumption, and the prevalence of self-reported peptic ulcer-related GI symptoms among a sample of 400 Jordanian adults. By providing insights into the prevalence and risk factors for peptic ulcers in this population, the findings of this study may inform targeted prevention and management strategies, ultimately contributing to reducing the burden of potential GI disorders.

## 2. Materials and Methods

### 2.1. Study Design and Population

This descriptive self-reported cross-sectional study comprised a group of students currently attending The Hashemite University and a group of participants from the general population in Jordan. Inclusion criteria were individuals aged 18 years and above, residing in Jordan, and willing to participate in the study. Exclusion criteria were individuals below 18 years of age, those residing outside of Jordan, and those unwilling to provide informed consent. This study utilized an online questionnaire delivered to participants over a period of one month, from 1 February 2023 to 1 March 2023. The online questionnaire was created using Google Forms and posted on several social and university-related online platforms and/or pages. A total of 400 participants were included in the study.

### 2.2. Tools

The online questionnaire consisted of three main sections: sociodemographics, knowledge and self-reported symptoms of PUD, and NSAID use and consumption of xanthine and xanthine derivatives (theophylline and caffeine). The questions were designed to gather comprehensive information about the participants’ characteristics, PUD-related symptoms, medication use, and dietary habits. The survey was developed by the research team specifically for this study, with the choice of questions based on a comprehensive review of the literature on related GI symptoms and their potential associations with NSAIDs use and caffeine consumption [1,4,5,6,7]. The questions were designed to capture a wide range of common GI symptoms, such as burning stomach pain, feeling of fullness, bloating, and heartburn, as well as less common but potentially serious symptoms like dark blood in stool and unexplained weight loss. Additionally, the questionnaire included items on the frequency and duration of these symptoms, as well as their relationship to food intake, to provide a more comprehensive understanding of the participants’ self-reported GI health. 

The questions were adapted from previously published surveys on GI symptoms, NSAID use and caffeine consumption, ensuring their relevance and validity [1,4,5,6,7]. The questions were reviewed by a panel of experts to ensure their relevance, clarity, and comprehensiveness. The survey was pilot-tested (*n* = 10 respondents), and proper modifications were made before administering it to all participants. The average time taken to complete the survey was approximately 10 min.

### 2.3. Data Management and Analysis

The survey data were exported from Google Forms into Microsoft Excel (version 2408) for data management. The data were checked for errors, inconsistencies, and missing values. Subsequently, the data were imported into SPSS version 23 for statistical analysis. Categorical variables were recoded into meaningful categories, while continuous variables were left unchanged. Descriptive statistics, including frequencies, percentages, means, and standard deviations, were used to summarize the data.

Contingency tables were constructed to calculate odds ratios (ORs) and their corresponding 95% confidence intervals (CIs) for the associations between NSAIDs usage, caffeinated drink consumption, and self-reported GI symptoms. NSAIDs usage was defined as consumption of any of the seven mentioned forms of NSAIDs, while caffeinated drinks usage was defined as consumption of tea, coffee, energy, or soft drinks. The chi-square test was used to examine the statistical significance of these associations. Subgroup analyses were performed by age and gender. A *p*-value of less than 0.05 was considered statistically significant.

### 2.4. Ethical Considerations

The study protocol was reviewed and approved by the Institutional Review Board (IRB) of The Hashemite University (approval number: 17/5/2022/2023). All participants provided informed consent before participating in the study. Participation was voluntary, and participants could withdraw from the study at any time without consequences. Personal identifiers were not collected to maintain participant confidentiality. The data were stored on password-protected computers accessible only to the research team.

## 3. Results

### 3.1. General Characteristics of Surveyed Responders

A total of 400 respondents were included in the final analysis. The majority of participants were young adults, with 65% (*n* = 262) aged 18–24 years and 20% (*n* = 79) aged 25–31 years. Only 15% (*n* = 59) of respondents were older than 32 years. The sample had a slightly higher proportion of females (59%, *n* = 235) compared to males (41%, *n* = 165). Most respondents were single (73%, n = 294), while 25% (n = 101) were married, and only a few were widowed (1%, *n* = 3) or divorced (1%, *n* = 2). Regarding the lifestyle factors, 25% (*n* = 98) of participants reported smoking, and only 2% (*n* = 8) consumed alcohol, making any impact on any of the observed GI symptoms likely to be minimal. These findings are summarized in Table 1.

### 3.2. Self-Reported Peptic Ulcer Disease (PUD) and Gastrointestinal (GI) Symptoms

The prevalence of self-reported PUD-related symptoms in our cohort was 6.0%. Participants were asked to report current GI symptoms through a series of eight questions (Table 2). The most commonly self-reported symptoms were heartburn (53.5%), feeling of fullness (50.8%), and bloating or belching (50.5%). Around one-third of participants reported burning stomach pain (29.5%) and recent changes in appetite (35.3%). Around one-fifth of respondents (20.5%) experienced intolerance to fatty food, while 9% suffered from unexplained weight loss.

Regarding the frequency of the experienced symptoms, 19.5% of respondents never suffered from any of the surveyed symptoms, while 45% and 9% experienced their symptoms occasionally or daily, respectively. Around 14.8% reported weekly symptoms, and 11.8% had monthly symptoms (Table 2). When asked about the duration of their symptoms, 7.3% of respondents reported that symptoms lasted for a few seconds, while 38.1% and 32.7% experienced symptoms lasting minutes and hours, respectively. Only 2.5% of participants reported continuous symptoms, and 19.5% had no symptoms at all. Nearly half of the respondents (47.8%) never suffered pain related to eating, while 11.8% reported decreased pain, 30.5% experienced increased pain, and 10% had constant pain (Table 2).

Regarding the association between self-reported symptoms and spicy food, 36% of participants reported no association, while 32.5% experienced increased symptoms with spicy food. A similar percentage (31.5%) were unsure about this association (Table 2).

In summary, the most commonly self-reported GI symptoms were heartburn, feeling of fullness, and bloating or belching, with around one-third (30.5%) associated with eating habits. The prevalence of self-reported PUD was 6.0%.

### 3.3. NSAIDs Used and Association with Self-Reported GI Symptoms

Among the 400 respondents, ibuprofen (47%) and diclofenac (23.8%) were the most commonly used NSAIDs (Table 3). Aspirin, well-known for its potential to contribute to GI ulcers, was reported to be used by 17.8% of participants. Indomethacin was the least commonly used agent, with only one person (0.3%) reporting its use. Personal experience and physician prescription were equally the most common ways to obtain NSAIDs, each reported by 42% of the respondents. In contrast, 16% of the participants said they used these agents based on advice from non-medical personnel. More than half of the participants (59.3%) believed they were knowledgeable about the side effect profile of NSAIDs.

When asked about the symptoms experienced post-NSAID use, the respondents most commonly reported abdominal discomfort (28.3%), abdominal pain (12.3%), and heartburn (12.3%). Other self-reported symptoms included acid reflux (8.5%), dyspepsia (5.5%), and upper GI bleeding (1.5%). Notably, 65.5% of the participants reported not suffering from any of the investigated symptoms (Table 3).

In conclusion, ibuprofen and diclofenac were the most commonly used NSAIDs among our study’s population, with personal experience and physician prescription being the most common ways to obtain them. Abdominal discomfort was the most frequently reported symptom post-NSAID consumption.

### 3.4. Correlation of Self-Reported GI Symptoms with Caffeinated Beverages

Coffee, tea, soft drinks, and energy drinks were consumed by 75.5%, 52.8%, 42.5%, and 16.3% of the sampled population, respectively (Table 4). Most participants (46%) reported drinking less than 1 cup/day of their favorite beverage, while 43.3% consumed 1 to 2 cups/day, and only 10.8% drank more than 3 cups/day. The majority of respondents (72.5%) consumed caffeinated drinks on a daily basis, while 7.3%, 14%, and 0.5% reported drinking these beverages occasionally, weekly, and monthly, respectively. Regarding the time of consumption, less than one-third (29.8%) of the participants drank during the morning, while 11.8% and 13.8% consumed their drinks during the afternoon and evening, respectively (Table 4). The remaining 44.8% reported drinking at any time of the day.

Among the post-caffeine consumption symptoms, stomach pain (29.8%), feeling of fullness (20.3%), bloating (20.3%), and troubled breathing (18.5%) were the most commonly experienced, while appetite change was the least common symptom, reported by only one participant (0.3%). Notably, more than half (51.3%) of the pooled sample mentioned not suffering from any of the questioned symptoms (Table 4). 

### 3.5. Impact of NSAIDs Usage on Self-Reported GI Symptoms

The impact of NSAIDs usage on GI symptoms was assessed using odds ratios (ORs) and 95% CIs (Table 5).The odds of being diagnosed with a peptic ulcer (based on self-reported past medical history) were 2.431 times higher among NSAIDs users (95% CI: 0.813–7.266), although this association was not statistically significant (*p* = 0.117). Post-NSAIDs usage, the odds of experiencing acid reflux (OR = 2.900, 95% CI: 1.095–7.678, *p* = 0.033), abdominal pain (OR = 4.688, 95% CI: 1.812–12.131, *p* < 0.001), discomfort (OR = 8.068, 95% CI: 3.926–16.583, *p* < 0.001), and heartburn (OR = 2.629, 95% CI: 1.194–5.787, *p* = 0.014) were significantly higher compared to non-users. The odds of experiencing dyspepsia were also higher among NSAIDs users (OR = 3.092, 95% CI: 0.898–10.646), but this association was not statistically significant (*p* = 0.063).

These findings suggest that NSAIDs usage is associated with an increased risk of various GI symptoms, with the most significant effects observed for abdominal pain and discomfort.

### 3.6. Correlation between Caffeine Consumption and Self-Reported GI Symptoms

The analysis of caffeine consumption revealed significant correlations with several GI symptoms (Table 6). The odds of being diagnosed with a peptic ulcer after caffeine usage were 1.994 times higher than non-users (95% CI: 0.260–15.284), but this association was not statistically significant (*p* = 0.498). However, substantial increases in the odds were noted for burning stomach pain (OR = 14.104, 95% CI: 1.900–104.655, *p* < 0.001), feeling of fullness (OR = 8.304, 95% CI: 1.115–61.836, *p* = 0.010), and bloating or belching (OR = 8.304, 95% CI: 1.115–61.836, *p* = 0.010) among caffeine consumers compared to non-consumers. The associations between caffeine consumption and heartburn, intolerance to fatty food, appetite changes, dark blood in stool, and unexplained weight loss could not be assessed due to insufficient data. The odds of experiencing trouble breathing were 1.580 times higher among caffeine consumers (95% CI: 0.536–4.662), but this association was not statistically significant (*p* = 0.480).

These findings indicate that caffeine intake (and NSAIDs to some extent) is significantly associated with an increased risk of severe GI symptoms such as burning stomach pain, fullness, and bloating, emphasizing the importance of moderation in caffeine consumption to prevent adverse health effects.

### 3.7. Associations between Smoking and Self-Reported GI Symptoms

Lastly, we explored the correlation between smoking habits and GI symptoms (Table 7). As expected, smoking was significantly associated with an increased risk of several GI symptoms, including burning stomach pain (OR = 2.998, *p* < 0.001), feeling of fullness (OR = 1.967, *p* = 0.004), bloating or belching (OR = 1.993, *p* = 0.004), heartburn (OR = 2.146, *p* = 0.002), and appetite changes (OR = 1.722, *p* = 0.021). However, smoking was not significantly associated with intolerance to fatty food, dark blood in stool, trouble breathing, or unexplained weight loss.

Regarding symptoms experienced after NSAIDs usage, smoking was significantly associated with an increased risk of discomfort (OR = 3.500, *p* < 0.001) but not with acid reflux, stomach ache, heartburn, dyspepsia, or self-reported upper or lower GI bleeding.

### 3.8. Clinical Associations between NSAIDs Use or Caffeine Consumption with the Diagnosis of Peptic Ulcers or Post-Consumption Symptoms

The OR analysis revealed that the consumption of caffeinated beverages is associated with various GI symptoms (Table 8 and Table 9). Coffee consumption showed a significant association with burning stomach pain (OR: 2.233, 95% CI: 1.270–3.927, *p* = 0.005), feeling of fullness (OR: 2.386, 95% CI: 1.207–4.719, *p* = 0.011), bloating or belching (OR: 2.386, 95% CI: 1.207–4.719, *p* = 0.011), heartburn (OR: 12.715, 95% CI: 1.719–94.080, *p* = 0.001), and trouble breathing (OR: 3.706, 95% CI: 1.640–8.376, *p* = 0.001) (Table 8).

Similarly, tea consumption was associated with burning stomach pain (OR: 2.349, 95% CI: 1.499–3.679, *p* < 0.001), feeling of fullness (OR: 2.946, 95% CI: 1.723–5.039, *p* < 0.001), bloating or belching (OR: 2.946, 95% CI: 1.723–5.039, *p* < 0.001), and heartburn (OR: 8.268, 95% CI: 2.866–23.855, *p* < 0.001) (Table 8). Energy drink consumption was linked to increased odds of burning stomach pain (OR: 2.193, 95% CI: 1.270–3.784, *p* = 0.004), feeling of fullness (OR: 4.384, 95% CI: 2.476–7.764, *p* < 0.001), bloating or belching (OR: 4.384, 95% CI: 2.476–7.764, *p* < 0.001), heartburn (OR: 5.890, 95% CI: 2.863–12.118, *p* < 0.001), appetite changes (OR: 4.157, 95% CI: 1.392–12.416, *p* = 0.015), dark blood in stool (OR: 7.257, 95% CI: 1.585–33.235, *p* = 0.011), and trouble breathing (OR: 2.539, 95% CI: 1.398–4.613, *p* = 0.003) (Table 9). Soft drink consumption was significantly associated with burning stomach pain (OR: 2.018, 95% CI: 1.307–3.115, *p* = 0.002), feeling of fullness (OR: 4.028, 95% CI: 2.385–6.803, *p* < 0.001), bloating or belching (OR: 4.028, 95% CI: 2.385–6.803, *p* < 0.001), heartburn (OR: 6.552, 95% CI: 2.795–15.359, *p* < 0.001), and trouble breathing (OR: 2.328, 95% CI: 1.391–3.896, *p* = 0.002) (Table 9).

The consumption of caffeinated beverages, including coffee, tea, energy drinks, and soft drinks was significantly associated with increased odds of various self-reported GI symptoms, with the strongest associations observed for heartburn with coffee (OR = 12.715, *p* = 0.001) and tea consumption (OR = 8.268, *p* < 0.001), and for feeling of fullness and bloating or belching with energy drink and soft drink consumption (ORs > 4.0, *p* < 0.001). These findings highlight a significant association between the consumption of caffeinated beverages and the increased self-reporting of GI discomfort, emphasizing the need for moderation in their intake to mitigate adverse health effects. The results suggest that individuals experiencing GI symptoms should consider reducing their intake of caffeinated beverages, particularly if they consume multiple types of these drinks.

## 4. Discussion

This cross-sectional study explored the association between the use of NSAIDs or caffeinated drinks with GI health and self-reported PUD-related symptoms in a cohort of 400 Jordanian individuals predominantly within the age range of 18–24 years. The prevalence of self-reported PUD symptoms in our cohort was around 6.0%, which aligns with global estimates [2]. Moreover, the high prevalence of GI symptoms reported by respondents, including heartburn (53.5%), bloating (50.5%), and discomfort (50.8%), underscores the burden of these conditions on individuals’ quality of life and healthcare systems [17]. These findings are consistent with previous studies highlighting the common occurrence of GI symptoms in the general population, particularly among younger age groups [18].

### 4.1. The Association between NSAIDs and GI Symptoms

The association between NSAIDs use and self-reported GI symptoms is a central finding of this study. Despite the widespread availability and use of NSAIDs for pain management, our results indicate a significant correlation between NSAIDs usage and self-reported symptoms such as discomfort, abdominal pain, and acid reflux [19]. These findings corroborate existing research demonstrating the GI risks associated with NSAIDs administration, emphasizing the need for cautious use and patient education regarding medication safety [19,20]. Although some of these symptoms, such as dyspepsia, are known side effects of NSAIDs [21], this association warrants further investigation, particularly in light of the 25% smoking prevalence among respondents, which is a known risk factor for GI diseases [22,23]. The increased odds of developing PUD among NSAIDs users, although not statistically significant (*p* = 0.117), further highlights the potential impact of these medications on GI health. The mechanisms underlying NSAID-induced GI injury involve the inhibition of COX enzymes, leading to reduced prostaglandin synthesis and impaired mucosal defense [24]. Additionally, direct injury and the generation of reactive oxygen species contribute to the development of erosions and ulcers [25].

### 4.2. Influence of Dietary and Geographic Factors

It is important to acknowledge that the GI symptoms reported in our study may be influenced by factors beyond NSAIDs use and caffeine consumption, such as food quality, dietary habits, and geographic location. The Jordanian diet, characterized by a high intake of vegetables, fruits, whole grains, and legumes, as well as the consumption of traditional dishes like mansaf and falafel [8,9], may contribute to the relatively higher prevalence of certain GI symptoms. Additionally, regional differences in food preparation methods, spice use, and food storage practices could impact the gut microbiome and GI health [10,11]. Future studies should aim to compare our findings with similar investigations conducted in other countries, considering the unique dietary and cultural factors that may influence GI symptom prevalence. Such comparisons could help elucidate the relative contributions of NSAIDs, caffeine, and other lifestyle factors to GI health across different populations.

### 4.3. Role of Dietary Factors

Interestingly, the survey also highlighted a potential link between spicy food consumption and GI symptoms. While 36% of the participants reported no increase in symptoms with spicy food, a similar percentage experienced exacerbated symptoms. This finding suggests that a subset of the surveyed population may have an increased sensitivity to dietary factors, which could interact with NSAID-induced GI irritation [26]. The role of dietary factors in the development and exacerbation of GI symptoms is complex, with studies suggesting that certain foods, such as those high in fat or spices, may increase the risk of dyspepsia and reflux [27]. However, the relationship between diet and GI health is highly individualized, and further research is needed to elucidate the specific mechanisms and potential interactions with medication use.

### 4.4. The Correlation between Caffeinated Beverages and GI Symptoms

Our study also sheds light on the correlation between caffeinated beverage consumption and GI symptoms. While moderate caffeine intake is generally considered safe, our findings suggest a significant association between caffeinated drinks and symptoms such as stomach pain, heartburn, fullness, and bloating. Coffee consumption was significantly associated with burning stomach pain, feeling of fullness, bloating or belching, and heartburn. Similarly, tea consumption was associated with these symptoms, with the strongest association found for heartburn. These findings are consistent with research suggesting caffeine’s role in relaxing the lower esophageal sphincter, potentially leading to gastroesophageal reflux [22,28].

A conclusive connection between the amount of caffeine and some GI symptoms such as heartburn has not been fully established previously. For example, a narrative review on the effects of coffee on the GI tract highlights that the relationship between coffee consumption and gastroesophageal reflux disease (GERD) is complex and not fully understood [12,13]. Some studies have found no significant correlation between coffee consumption and symptoms of GERD, while others suggest that coffee may either decrease or increase the risk of GERD, depending on factors such as the timing of coffee consumption and individual tolerance. Given these inconsistencies, it is clear that the relationship between coffee consumption and gastrointestinal symptoms like heartburn requires further investigation. Our study contributes to this ongoing discussion by providing evidence from a specific population, but we recognize the need for more comprehensive and longitudinal studies to fully understand this association.

Energy drinks and soft drinks were also linked to increased odds of these symptoms, particularly the feeling of fullness and bloating or belching, with ORs exceeding 4.0 and *p*-values less than 0.001. The high sugar content and carbonation of energy drinks and soft drinks may contribute to delayed gastric emptying and increased gas production, exacerbating symptoms such as the feeling of fullness and bloating [29]. Nettleton et al. reported that caffeine is a stimulant that increases gut motility, potentially leading to loose stools or diarrhea [28]. These results underscore the importance of moderation in caffeine consumption and highlight the potential health implications of excessive intake, particularly in individuals predisposed to GI disorders.

Trouble breathing, a symptom not typically associated with GI disorders, was significantly correlated with all caffeinated beverages, most strongly with coffee. This finding may be due to caffeine’s systemic effects, including its impact on the respiratory system, as suggested by a recent clinical study reporting that caffeine consumption alters the breathing pattern by increasing tidal volume and lengthening the inspiratory phase of the respiratory cycle [30]. The potential mechanisms linking caffeine intake to respiratory symptoms warrant further investigation.

### 4.5. Smoking and GI Symptoms

Our analysis of the data stratified by smoking status revealed significant associations between smoking and several GI symptoms, including burning stomach pain, feeling of fullness, bloating or belching, heartburn, appetite changes, feeling faint, nausea, and vomiting. These findings are consistent with previous studies that have identified smoking as a risk factor for various GI disorders [14,15]. Interestingly, smoking was also associated with an increased risk of discomfort after NSAIDs usage, suggesting a potential interaction between these two risk factors. The mechanisms underlying the association between smoking and gastrointestinal symptoms are complex and may involve alterations in gut motility, increased acid secretion, and impaired mucosal defense.

### 4.6. Limitations and Future Directions

It is important to acknowledge the limitations of this study, including its cross-sectional design, which precludes causal inference, and reliance on self-reported data, which may introduce recall bias. The representativeness of the sample, particularly the predominance of young adults, may limit the generalizability of the findings to other age groups. Additionally, the lack of detailed information on the duration and dosage of NSAIDs and caffeine consumption hinders the assessment of potential dose–response relationships.

In terms of the study instrument’s design, some of the questions might not have captured the intended information with the highest level of specificity or accuracy. For example, asking about the presence of certain symptoms such as the presence of dark blood in stool or differentiating acid reflux without confirmatory diagnostic procedures could lead to misinterpretation or misclassification. Additionally, questions regarding caffeine consumption might not have accounted for all sources of caffeine or the possibility of decaffeinated beverages. Another limitation is the combination of bloating and belching into a single question in our survey. Bloating and belching are distinct symptoms with different pathophysiological mechanisms [16], and assessing them separately would have provided a more accurate representation of their prevalence and associations with NSAIDs use and caffeine consumption. Moreover, we did not collect detailed information on other medications or medical conditions that could influence GI health, potentially confounding the observed associations.

Despite these limitations, our study provides valuable insights into the associations between NSAIDs use, caffeine consumption, and GI health in a young adult population, highlighting the need for further research to elucidate the underlying mechanisms, identify at-risk populations, and develop targeted interventions.

## 5. Conclusions

In this cross-sectional study, we investigated the associations between NSAIDs use, caffeine consumption, and self-reported GI symptoms in a subpopulation of young Jordanian adults. Our findings demonstrate that NSAIDs use and caffeine consumption were independently associated with increased risks of various GI symptoms, including stomachache, discomfort, acid reflux, and heartburn. The consumption of specific caffeinated beverages, such as coffee, tea, soft drinks, and energy drinks, was also associated with increased odds of multiple GI symptoms. These findings highlight the importance of considering the potential GI effects of NSAIDs and caffeine, particularly when consumed in combination. Healthcare professionals should be aware of these associations when managing patients with GI complaints and provide appropriate guidance on the safe use of NSAIDs and moderation of caffeine intake.

## Figures and Tables

**Table 1:** General characteristics of the sample that were surveyed and included in the final analysis.

Characteristic		*N* = 400
Age	18–24	262 (65%)
	25–31	79 (20%)
	32–38	18 (4%)
	39–45	14 (4%)
	46–52	11 (3%)
	53–59	9 (2%)
	60–65	7 (2%)
Gender	Female	235 (59%)
	Male	165 (41%)
Marital status	Single	294 (73%)
	Married	101 (25%)
	Widowed	3 (1%)
	Divorced	2 (1%)
Smoker	No	302 (75%)
	Yes	98 (25%)
Alcohol	No	392 (98%)
	Yes	8 (2%)

**Table 2:** The association between gastrointestinal (GI) symptoms in the final sample included for analysis.

Question	Answer	*N*=	%
Have you ever been diagnosed with peptic ulcer?	No	376	94.0%
	Yes	24	6.0%
Do you feel burning stomach pain?	No	282	70.5%
	Yes	118	29.5%
Do you experience feeling of fullness?	No	197	49.3%
	Yes	203	50.8%
Do you have bloating or belching?	No	198	49.5%
	Yes	202	50.5%
Do you experience heartburn?	No	186	46.5%
	Yes	214	53.5%
Are there any appetite changes?	No	259	64.8%
	Yes	141	35.3%
Do you think you have intolerance to fatty food?	No	318	79.5%
	Yes	82	20.5%
Have you ever noticed dark blood in stool?	No	389	97.3%
	Yes	11	2.8%
Have you had unexplained weight loss?	No	364	91.0%
	Yes	36	9.0%
How often do you experience these symptoms?	Never	78	19.5%
	Occasionally	180	45.0%
	Daily	36	9.0%
	Weekly	59	14.8%
	Monthly	47	11.8%
How long do your symptoms last?	Never	78	19.5%
	Seconds	29	7.3%
	Minutes	152	38.1%
	Hours	171	32.7%
	Continuous	10	2.5%
Is pain affected by eating?	No pain	191	47.8%
	Decreased	47	11.8%
	Increased	122	30.5%
	Constant	40	10%
Do symptoms increase with spicy foods?	No	144	36%
	Yes	130	32.5%
	Maybe	126	31.5%

**Table 3:** NSAID use in the sample cohort and its association with GI symptoms.

		*N*	%
Aspirin	No	329	82.3%
	Yes	71	17.8%
Celecoxib	No	386	96.5%
	Yes	14	3.5%
Diclofenac	No	305	76.3%
	Yes	95	23.8%
Mefenamic acid	No	392	98%
	Yes	8	2%
Indomethacin	No	399	99.8%
	Yes	1	0.3%
Naproxen	No	348	87%
	Yes	52	13%
Ibuprofen	No	212	53%
	Yes	188	47%
None	No	271	67.8%
	Yes	129	32.3%
Do you use NSAIDs constantly?	No	357	89.3%
	Yes	43	10.8%
How do you buy NSAIDs?	Non-medical advice	64	16%
	Personal experience	168	42%
	Prescription	167	42%
Do you have knowledge about NSAID side effects?	No	163	40.8%
	Yes	237	59.3%
Have you ever experienced acid reflux after NSAID use?	No	366	91.5%
	Yes	34	8.5%
Have you ever experienced abdominal pain after NSAID use?	No	351	87.8%
	Yes	49	12.3%
Have you ever experienced discomfort after NSAID use?	No	287	71.8%
	Yes	113	28.3%
Have you ever experienced heartburn after NSAID use?	No	351	87.8%
	Yes	49	12.3%
Have you ever experienced dyspepsia after NSAID use?	No	378	94.5%
	Yes	22	5.5%
Have you ever experienced upper or lower GI bleeding after NSAID use?	No	394	98.5%
	Yes	6	1.5%
Have you never experienced any symptoms after NSAID use?	No	138	34.5%
	Yes	262	65.5%

**Table 4:** The relationship between caffeinated drinks and GI symptoms.

		*N*	%
Do you drink coffee?	No	98	24.5%
	Yes	302	75.5%
Do you drink tea?	No	189	47.3%
	Yes	211	52.8%
Do you drink energy drinks?	No	335	83.8%
	Yes	65	16.3%
Do you drink soft drinks?	No	230	57.5%
	Yes	170	42.5%
None	No	362	90.5%
	Yes	38	9.5%
How much do you drink?	Less than 1 cup	184	46.0%
	1 to 2 cups	173	43.3%
	More than 3 cups	43	10.8%
How often do you drink the aforementioned?	Never	23	5.8%
	Occasionally	29	7.3%
	Daily	290	72.5%
	Weekly	56	14.0%
	Monthly	2	0.5%
When do you consume the most caffeine?	Morning	119	29.8%
	Afternoon	47	11.8%
	Evening	55	13.8%
	Anytime	179	44.8%
Do you experience burning stomach pain when consuming any of the drinks?	No	281	70.3%
	Yes	119	29.8%
Do you experience feeling of fullness when consuming any of the drinks?	No	319	79.8%
	Yes	81	20.3%
Do you experience bloating or belching when consuming any of the drinks?	No	319	79.8%
	Yes	81	20.3%
Do you experience heartburn when consuming any of the drinks?	No	364	91.0%
	Yes	36	9.0%
Do you experience appetite changes when consuming any of the drinks?	No	399	99.8%
	Yes	1	0.3%
Do you experience intolerance to fatty foods when consuming any of the drinks?	No	386	96.5%
	Yes	14	3.5%
Do you notice dark blood in stool when consuming any of the drinks?	No	393	98.3%
	Yes	7	1.8%
Do you experience trouble breathing when consuming any of the drinks?	No	326	81.5%
	Yes	74	18.5%
Have you never experienced any symptoms after consuming any of these drinks?	No	195	48.8%
	Yes	205	51.3%

**Table 5:** Risk of developing PUD and/or GI symptoms from using NSAIDs. Significant *p*-values are presented in bold to highlight statistically significant results.

	OR	LL	UL	*p*-Value
Prevalence				
Diagnosed with peptic ulcer	2.431	0.813	7.266	0.117
Symptoms post-NSAID usage				
Acid reflux	2.900	1.095	7.678	**0.033**
Abdominal pain	4.688	1.812	12.131	**>0.001**
Discomfort	8.068	3.926	16.583	**>0.001**
Heartburn	2.629	1.194	5.787	**0.014**
Dyspepsia	3.092	0.898	10.646	0.063
Upper or lower GI bleeding *	NA	NA	NA	NA

* This indicates irrelevant statistical associations due to violation of statistical assumptions. OR: odds ratio; LL: lower limit of confidence interval; UL: upper limit of confidence interval.

**Table 6:** Risk of developing PUD and/or GI symptoms from consuming caffeinated drinks. Significant *p*-values are presented in bold to highlight statistically significant results.

	OR	LL	UL	*p*-Value
Prevalence				
Diagnosed with peptic ulcer	1.994	0.260	15.284	0.498
Symptoms post-caffeine usage				
Burning stomach pain	14.104	1.900	104.655	**>0.001**
Feeling of fullness	8.304	1.115	61.836	**0.010**
Bloating or belching	8.304	1.115	61.836	**0.010**
Heartburn *	NA	NA	NA	NA
Appetite changes *	NA	NA	NA	NA
Intolerance to fatty food *	NA	NA	NA	NA
Dark blood in stool *	NA	NA	NA	NA
Trouble breathing	1.580	0.536	4.662	0.480
Unexplained weight loss *	NA	NA	NA	NA

* This indicates irrelevant statistical associations due to violation of statistical assumptions. OR: odds ratio; LL: lower limit of confidence interval; UL: upper limit of confidence interval.

**Table 7:** Risk of developing GI symptoms from smoking.

	OR	LL	UL	*p*-Value
Have you had these symptoms?				
Burning stomach pain	2.998	1.860	4.832	0.000
Feeling of fullness	1.967	1.231	3.143	0.004
Bloating or belching	1.993	1.247	3.185	0.004
Heartburn	2.146	1.330	3.464	0.002
Appetite changes	1.722	1.081	2.743	0.021
Intolerance to fatty food	1.364	0.793	2.346	0.260
Dark blood in stool *	NA	NA	NA	NA
Trouble breathing	1.292	0.725	2.302	0.383
Unexplained weight loss	0.724	0.307	1.709	0.460
Symptoms post-NSAID usage				
Acid reflux	1.534	0.719	3.272	0.266
Abdominal pain	1.271	0.653	2.476	0.479
Discomfort	3.500	2.162	5.667	0.000
Heartburn	1.771	0.935	3.353	0.077
Dyspepsia	1.829	0.743	4.499	0.183
Upper or lower GI bleeding *	NA	NA	NA	NA

* This indicates irrelevant statistical associations due to violation of statistical assumptions. OR: odds ratio; LL: lower limit of confidence interval; UL: upper limit of confidence interval.

**Table 8:** Statistical associations between coffee or tea and post-drinking GI symptoms. Significant *p*-values are presented in bold to highlight statistically significant results.

	Coffee				Tea			
	OR	LL	UL	*p*	OR	LL	UL	*p*
Burning stomach pain	2.233	1.270	3.927	**0.005**	2.349	1.499	3.679	**>0.001**
Feeling of fullness	2.386	1.207	4.719	**0.009**	2.946	1.723	5.039	**>0.001**
Bloating or belching	2.386	1.207	4.719	**0.011**	2.946	1.723	5.039	**>0.001**
Heartburn	12.715	1.719	94.080	**0.001**	8.268	2.866	23.855	**>0.001**
Intolerance to fatty food *	0.805	0.247	2.626	0.718	1.640	0.540	4.982	0.426
Dark blood in stool *	1.966	0.234	16.536	0.526	1.198	0.265	5.423	0.814
Trouble breathing	3.706	1.640	8.376	**0.001**	1.603	0.955	2.690	0.072

* This indicates irrelevant statistical associations due to violation of statistical assumptions except for energy drinks; appetite change was removed because it was irrelevant for all types.

**Table 9:** Statistical associations between energy or soft drinks and post-drinking GI symptoms. Significant *p*-values are presented in bold to highlight statistically significant results.

	Energy Drinks				Soft Drinks			
	OR	LL	UL	*p*	OR	LL	UL	*p*
Burning stomach pain	2.193	1.271	3.784	**0.007**	2.018	1.307	3.115	**0.002**
Feeling of fullness	4.384	2.476	7.764	**>0.001**	4.028	2.385	6.803	**>0.001**
Bloating or belching	4.384	2.476	7.764	**>0.001**	4.028	2.385	6.803	**>0.001**
Heartburn	5.890	2.863	12.118	**>0.001**	6.552	2.795	15.359	**>0.001**
Intolerance to fatty food *	4.157	1.392	12.416	**0.015**	2.516	0.828	7.647	0.105
Dark blood in stool *	7.257	1.585	33.235	**0.015**	3.455	0.662	18.025	0.140
Trouble breathing	2.539	1.398	4.613	**0.003**	2.328	1.391	3.896	**0.002**

* This indicates irrelevant statistical associations due to violation of statistical assumptions, except for energy drinks; appetite change was removed because it was irrelevant for all types.

## Data Availability

The data that support the findings of this study are not publicly available due to the need to maintain participant confidentiality. The data may be made available from the corresponding author upon reasonable request.
